# The safety and efficacy of BCG combined with mitomycin C compared with BCG monotherapy in patients with non-muscle-invasive bladder cancer: A systematic review and meta-analysis

**DOI:** 10.1515/med-2024-1134

**Published:** 2025-03-26

**Authors:** Jianping Liu, Weijian Zhou, Wei Zhang, Congwang Chang, Peng Zhang, Guanghua Fu

**Affiliations:** Department of Urology, The First People’s Hospital of Yibin, Yibin, 644000, China; Clinical Medical Department, Weifang Medical University, Weifang, 261042, China

**Keywords:** urinary bladder neoplasms, non-muscle-invasive bladder cancer, intravesical therapies, Bacillus Calmette-Guérin, mitomycin C

## Abstract

**Introduction:**

We sought to determine the efficacy and safety of Bacillus Calmette-Guérin (BCG) combined with mitomycin C (MMC) compared with BCG monotherapy in intravesical therapies for non-muscle-invasive bladder cancer (NMIBC).

**Methods:**

We followed the recommended PRISMA guidelines for systematic reviews. Systematic literatures were performed on PubMed, EMBASE, Cochrane Library, CNKI, CBM, VIP, Wan Fang, and Clinical Trials.gov. Randomized controlled trials (RCTs) comparing BCG combined with MMC and BCG monotherapy in intravesical therapies for non-muscle-invasive bladder cancer patients were searched until August 1, 2023.

**Results:**

This meta-analysis included 11 RCTs with a total of 1,349 subjects. Compared with BCG monotherapy, BCG combined with MMC was associated with lower disease recurrence rate (relative risk [RR] 0.66, 95% confidence interval [CI]: 0.56–0.77, *P* < 0.00001), disease progression rate (RR 0.61, 95% CI: 0.44–0.84, *P* = 0.003), and disease-specific mortality (RR 0.46, 95% CI: 0.26–0.78, *P* = 0.004). However, there was a higher incidence of systemic adverse reactions (RR 1.57, 95% CI: 1.22–2.02, *P* = 0.0004). There was no significant difference in the incidence of local adverse reactions (RR 1.07, 95% CI: 0.95–1.20, *P* = 0.26) and all-cause mortality (RR 0.80, 95% CI: 0.62–1.03, *P* = 0.08) between the two groups.

**Conclusions:**

BCG combined with MMC was associated with a decreased risk of bladder cancer recurrence and disease progression compared with BCG monotherapy. However, there was no significant difference in the incidence of local adverse events and all-cause mortality between the two groups. Due to the limitations of the number and quality of the included studies, more high-quality RCTs are needed to further explore the efficacy and safety of combined therapies.

## 1 Introduction

Bladder cancer is one of the most common malignancies of the genitourinary system, with the tenth highest incidence of all cancers worldwide, the sixth highest incidence of male malignancies, and the ninth highest mortality rate. According to cancer statistics in 2021, there were 573,000 new cases of bladder cancer worldwide in 2020, with 213,000 deaths, and the incidence of bladder cancer in men was generally higher than that in women. It is more common in men than in women, with respective incidence and mortality rates of 9.5 and 3.3 per 100,000 among men. Southern Europe has the highest incidence of bladder cancer in the world [1].

Non-muscle-invasive bladder cancer (NMIBC) accounts for about 75% of patients initially diagnosed with bladder cancer, including Ta, T1, and carcinoma *in situ* (Tis), among which Ta accounted for about 70%, T1 for about 20%, and Tis for about 10% [2]. NMIBC patients were divided into a low-risk group, an intermediate-risk group, a high-risk group, and a very high-risk group [3]. The low-risk group met the following conditions: a primary, single, Ta/T1 LG/G1 tumor <3 cm in diameter without *CIS* in a patient aged ≤70 years and a primary Ta LG/G1 tumor without *CIS* with at most ONE additional clinical risk factors (age >70 years, multiple papillary tumors, and tumor diameter ≥3 cm). The high-risk group met the following conditions: all T1 HG/G3 without *CIS* or all *CIS* patients, except those included in the very high-risk group. The very high-risk group met the following conditions: Ta HG/G3 and *CIS* with all three risk factors, T1 G2 and *CIS* with at least two risk factors, T1 HG/G3 and *CIS* with at least one risk factor, and T1 HG/G3 no *CIS* with all three risk factors. The intermediate risk group was the patients without *CIS* who were not included in either the low-, high-, or very high-risk groups [4].

Transurethral resection of bladder tumor (TURBT) is the standard treatment for NMIBC patients. However, due to the high recurrence and possible progression of the disease, postoperative adjuvant therapy is indispensable. The current guidelines recommend immediate intravesical infusion chemotherapy within 24 h after TURBT, as well as follow-up chemotherapy or immunotherapy based on pathological results. The standard treatment regimen for low-risk patients who need only one immediate perfusion and cannot be perfused immediately is still controversial. In patients with intermediate- and high-risk tumors, intravesical BCG after TURBT is more effective than TURB alone or TURB and intravesical chemotherapy [4]. However, some studies have shown better efficacy in combination therapy and should be given more attention.

Sylvester’s study has shown that many chemotherapy drugs, such as mitomycin C (MMC), epirubicin, and pirarubicin, have shown good efficacy, and the 5-year recurrence rate has been reduced by 14% compared with TURBT alone [5]. Since Morales A first used Bacillus Calmette-Guérin (BCG) in bladder instillation and put forward a 6-week induced instillation therapy in 1976 [6], a large number of studies have shown that BCG is superior to any other single drug in preventing disease recurrence, but the side effects caused by BCG infusion are also significantly more than those caused by chemotherapy [7].

Although BCG showed a good therapeutic effect, some patients still experience recurrence or progression after BCG treatment, so more attention and research are needed [8]. Radical cystectomy is the gold standard treatment option for BCG failure, while other available conservative treatments are thought to be oncologically poor [4]. For patients with poor overall health, conservative management is recommended to mitigate the potential complications of major surgical procedures. Nonetheless, there remains a lack of standardized treatment protocols for intravesical therapy [9].

Rajala’s study showed that BCG combined with MMC had stronger anti-tumor activity against bladder cancer cells than the BCG group, and there may be a synergistic effect between the two groups [10]. Therefore, BCG combined with MMC may be a potential choice to enhance the efficacy without increasing or even reducing the incidence of adverse reactions. Given the discrepancy between clinical practice and guidelines and uncertainty regarding optimal treatment strategies, we conducted a meta-analysis on the benefits and harms of intravesical therapies about BCG combined with MMC or BCG alone for NMIBC.

## 2 Materials and methods

This study carries on the analysis report according to the Preferred Reporting Items for Systematic Reviews and Meta-Analyses (PRISMA) system review and meta-analysis [11].

### 2.1 Study search and selection

Through a systematic literature search of PubMed, EMBASE, Cochrane Library, CNKI, CBM, VIP, Wan Fang, and Clinical Trials.gov, we found the related randomized controlled trials (RCTs) of BCG combined with MMC intravesical instillation in the treatment of NMIBC patients compared with BCG alone. The search time limit is from the establishment of the database to August 1, 2023. Each database is searched systematically according to the established retrieval strategy, and the search results are imported into the document management software EndNote. Titles and abstracts were examined by two independent reviewers according to the eligibility criteria. If there are differences in the results of the two researchers during the screening process, discuss or invite the third researcher to help solve the problem.

### 2.2 Eligibility criteria

Studies were included if they met the following criteria: (1) participants: all patients were pathologically diagnosed as NMIBC for the first time; (2) intervention: intravesical instillation of BCG combined with MMC; (3) control: intravesical instillation of BCG alone; (4) outcomes: at least one of the following, disease recurrence, disease progression, adverse reactions, disease-specific mortality, all-cause death; and (5) only RCTs were included. Studies were excluded if they met the following criteria: (1) the repeatedly published literature; (2) the studies compared the differences between BCG and MMC combination therapy with other agents; and (3) literature review, animal experiments, case reports, meta-analysis, etc.

### 2.3 Quality assessment and data extraction

Two researchers evaluated the quality of each original study with The Cochrane Collaboration Risk of Bias Tool, which included the following six items: (1) the generation of random sequences; (2) Allocation concealment; (3) whether to implement a blind method for participants and researchers; (4) whether to implement the blind method for outcome evaluation; (5) whether to selectively report research results; and (6) other sources of bias. The evaluation results are low risk, uncertain risk, and high risk. Two researchers independently evaluated the quality of the original research, and if there were inconsistencies in the evaluation process, they resolved their differences through discussion and finally reached an agreement.

Two researchers independently extracted the following data information: name of the first author, country, year of publication, type of study, sample size of the test group and the control group, average age, sex ratio, tumor stage, grade and risk stratification, follow-up time, dose, course of treatment and times of administration of BCG and MMC in the test group, dose and course of treatment of BCG in the control group, etc. The outcomes include recurrence rate, progression rate, adverse reactions (divided into local adverse reactions and systemic adverse reactions), all-cause mortality, and disease-specific mortality.

### 2.4 Statistical analysis

The meta-analysis of the original research data was carried out by using Revman5.4 software and Stata16.0 software. The main results of the study were tumor recurrence and disease progression after treatment during the follow-up period of each original study, and the secondary results were adverse reactions (local adverse reactions include hematuria, bladder irritation, chemical cystitis, systemic adverse reactions include fever, flu-like symptoms, etc.), all-cause mortality and disease-specific mortality.

The data types extracted in this study are binary variables, using relative risk (RR) and its 95% confidence interval (CI) to combine statistics. The forest Chi-square test and the size of *I* ^2^ were used to evaluate the heterogeneity among the included results. The size of heterogeneity is determined according to the value of *I* ^2^. When *P* ≥ 0.1 and *I* ^2^ < 50%, the heterogeneity among the studies is low, then the fixed effect model (FEM) is used for combined statistical analysis. When *P* < 0.1, *I* ^2^ > 50%, it indicates that the heterogeneity among the studies is large and then sensitivity analysis and subgroup analysis are used to find the source of heterogeneity and eliminate heterogeneity as far as possible. After excluding the influence caused by obvious clinical heterogeneity, the random effect model is selected for combined statistical analysis. The test level set by meta-analysis was *α* = 0.05 (*P* < 0.05), which indicated that the difference of combined statistical effect was statistically significant. Publication bias was detected by funnel chart, Egger regression analysis, and Begg rank correlation method.


**Ethical approval:** An ethics statement is not applicable because this study is based exclusively on published literature.

## 3 Results

### 3.1 Study selection and characteristics

**Figure 1:**
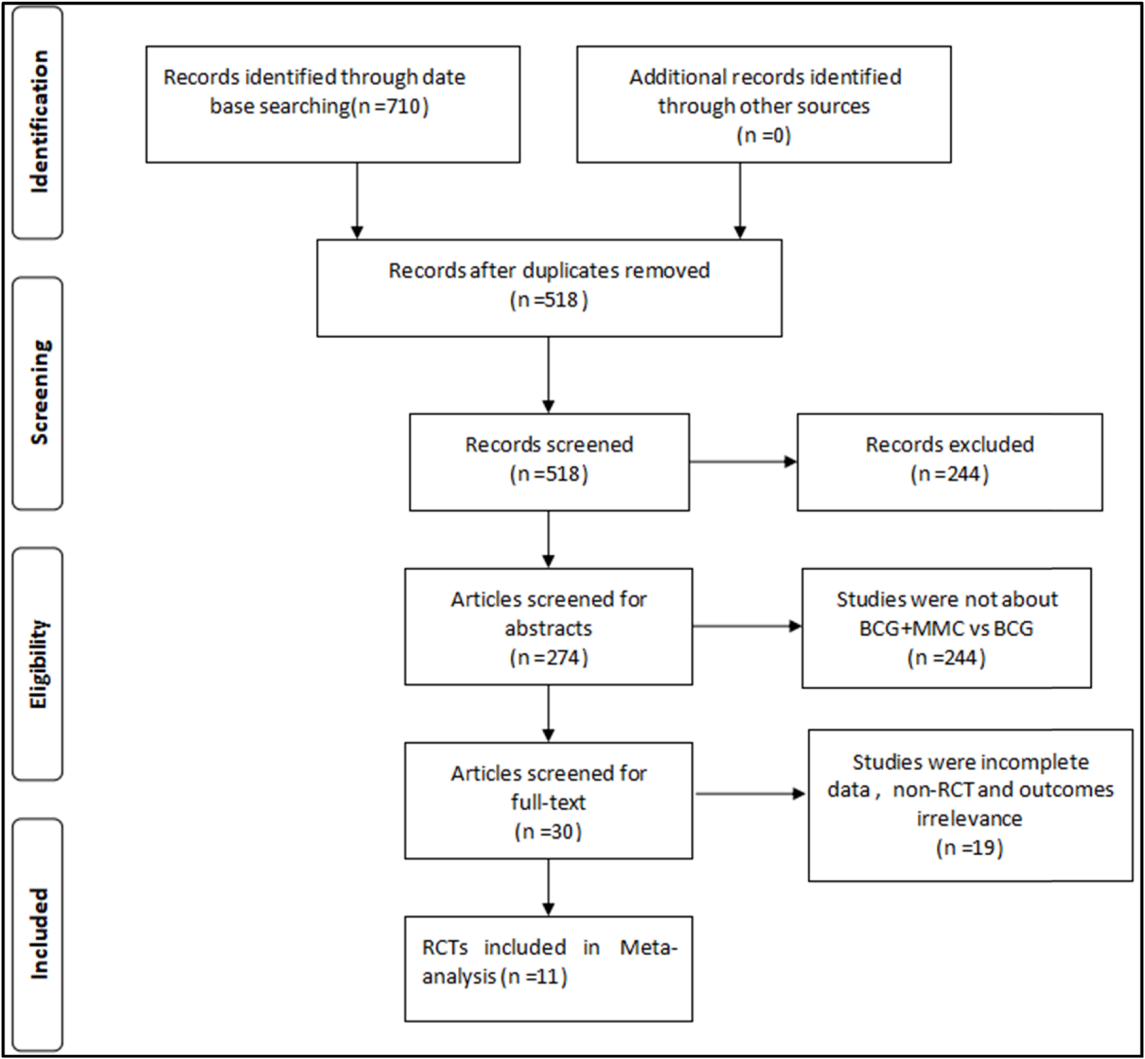
Flow diagram of studies’ selection process.

Through preliminary retrieval, a set of 710 articles was obtained, and 192 duplicated studies were first removed. Finally, 11 RCTs including 1,349 patients [12,13,14,15,16,17,18,19,20,21,22] were selected using the strategy shown in Figure 1. The characteristics of the included studies are summarized in Table 1. The sample size of each study ranged from 41 to 407, the average age of the subjects was 54–73 years old, and the average follow-up time ranged from 1 to 7.1 years. In three studies, only induced perfusion was performed without maintenance perfusion, and maintenance perfusion therapy was performed in the other eight studies. All eleven studies compared the outcome index of disease recurrence, six studies compared the outcome index of disease progression, eight studies compared local adverse reactions, and five studies compared systemic adverse reactions.

**Table 1:** Characteristics of included trials

Study and year	Country	Period	Simple size (M/F)	Mean age	stage	Follow-up	Treatment schedules (intervention)	Treatment schedules (control)
Di Stasi et al., 2006 [12]	Italy	1994–2002	212 (173/39)	66	T1, Tis	88 (63–110) m	BCG 81 mg/w, BCG 81 mg/w, MMC 40 mg/w (3 cycles) + MMC 40 mg/m, MMC 40 mg/m, BCG 81 mg/m (3 cycles)	BCG 81 mg/w, 6 w + BCG 81 mg/m, 10 m
Oosterlinck et al., 2011 [13]	Belgium	2001–2005	96 (86/9)1	68	Ta, T1, Tis	4.7 y	MMC 40 mg/w, 6 w + BCG 5 × 10^8^ CFU/w, 6 w + (MMC 40 mg/w, 1 w + BCG5 × 10^8^ CFU/w, 2 w) every 6 m until 3 y	BCG 5 × 10^8^ CFU/w, 9 w + (BCG5 × 10^8^ CFU/w, 3 w) every 6 m until 3 y
Gülpinar et al., 2012 [14]	Turkey	2004–2006	51 (41/10)	58	Ta, T1, Tis	41.3 (8–64) m	MMC 40 mg/w, 1 w + BCG 5 × 10^8^ CFU/w, 6 w	BCG 5 × 10^8^ CFU/w, 6 w
Solsona et al., 2015 [15]	Spain	1993–1994	407 (366/41)	65	Ta, T1, Tis	7.1 y	MMC 30 mg once + BCG 81 mg/w, 9 w	BCG 81 mg/w, 9 w
Chen et al., 2016 [16]	China		56			2 y	MMC 40 mg once + BCG 100 mg/w, 12 w	BCG 100 mg/w, 12 w
Fang et al., 2007 [17]	China	2000–2005	74 (63/11)	62	Ta, T1	6–36 m	MMC 40 mg/w, 6 w + BCG 120 mg, 6 w BCG 120 mg/m, 24 m	BCG 120 mg/w, 8 w
								BCG 120 mg/m, 24 m
Gong et al., 2014 [18]	China	2008–2010	95 (79/16)	59	Ta, T1	3 y	alternating MMC 40 mg/w, BCG 80 mg/w, 8 w + MMC 40 mg/2 w, BCG 80 mg/2 w, 3 m + MMC 40 mg/m, BCG 80 mg/m, 2 y	BCG 80 mg/w, 8 w + BCG 80 mg/2 w, 3 m + BCG 80 mg/m, 2 y
He et al., 2013 [19]	China	2005–2009	79 (62/17)	61	Ta, T1	21.2 ± 9.6 m	MMC 40 mg/w, 3 w + BCG 1 × 10^6^ CFU/w, 6 w + BCG 1 × 10^6^ CFU/m, 12 m + BCG1 × 10^6^ CFU/3 m, 9 m	BCG 1 × 10^6^ CFU/w, 6 w + BCG 1 × 10^6^ CFU/m, 12 m + BCG1 × 10^6^ CFU/3 m, 9 m
Liu and Liu, 2007 [20]	China	2000–2003	110 (84/26)	55	Ta, T1	35 (12–70) m	MMC 20 mg/w, 3 w + BCG 150 mg, 6 w	BCG 150 mg, 6 w + BCG 150 mg/w, 3 w at 3, 6, 12, 18, 24, 30, 36 m
							BCG 150 mg/w, 3 w at 3, 6, 12, 18, 24, 30, 36 m	
Abd El Kader, 2010 [21]	Egypt	2004–2008	128 (86/42)	54	Ta, T1	26 (6–45) m	MMC 40 mg/w, 1 w + BCG/w, 6 w	BCG/w, 6 w
Gao, 2018 [22]	China	2014–2016	41 (21/20)	68		1 y	alternating BCG 120 mg/w, MMC 40 mg/w, 8 w + BCG 120 mg/m, MMC 40 mg/m, 10 m	BCG 120 mg/w, 8 w + BCG 120 mg/m, 10 m

Abbreviations: BCG: Bacillus Calmette-Guérin; MMC: mitomycin C; CFU: colony-forming units; M: male; F: female; w: week; m: months; y: year.

### 3.2 Risk of bias in included studies

**Figure 2:**
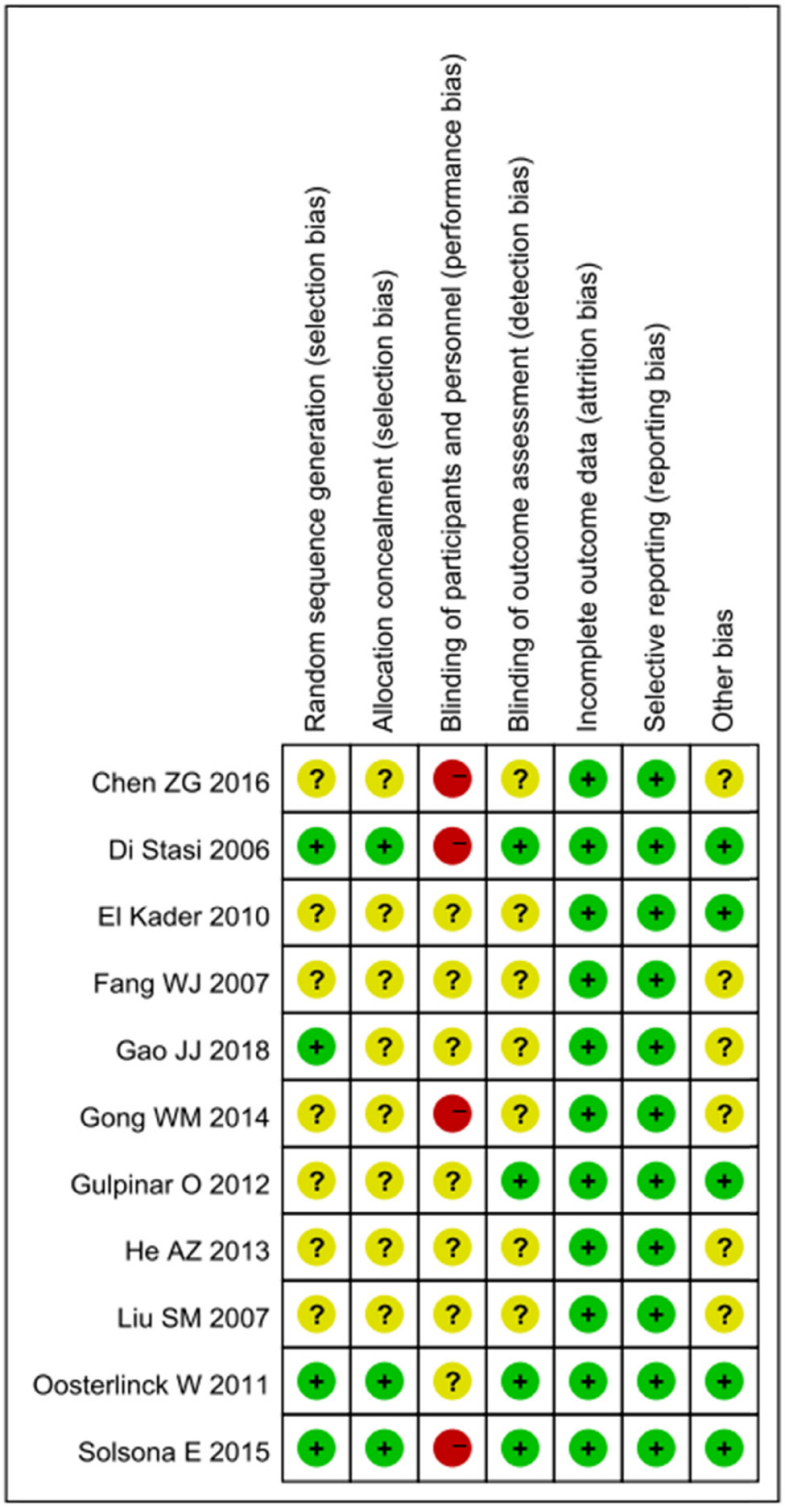
Assessment of risk of bias.

The results of quality assessment using the Cochrane Collaboration’s recommended tool. The quality assessment indicated that as a whole, risk of the selection bias, detection bias, and attrition bias were low in all the included studies (Figure 2).

### 3.3 Primary outcomes

**Figure 3:**
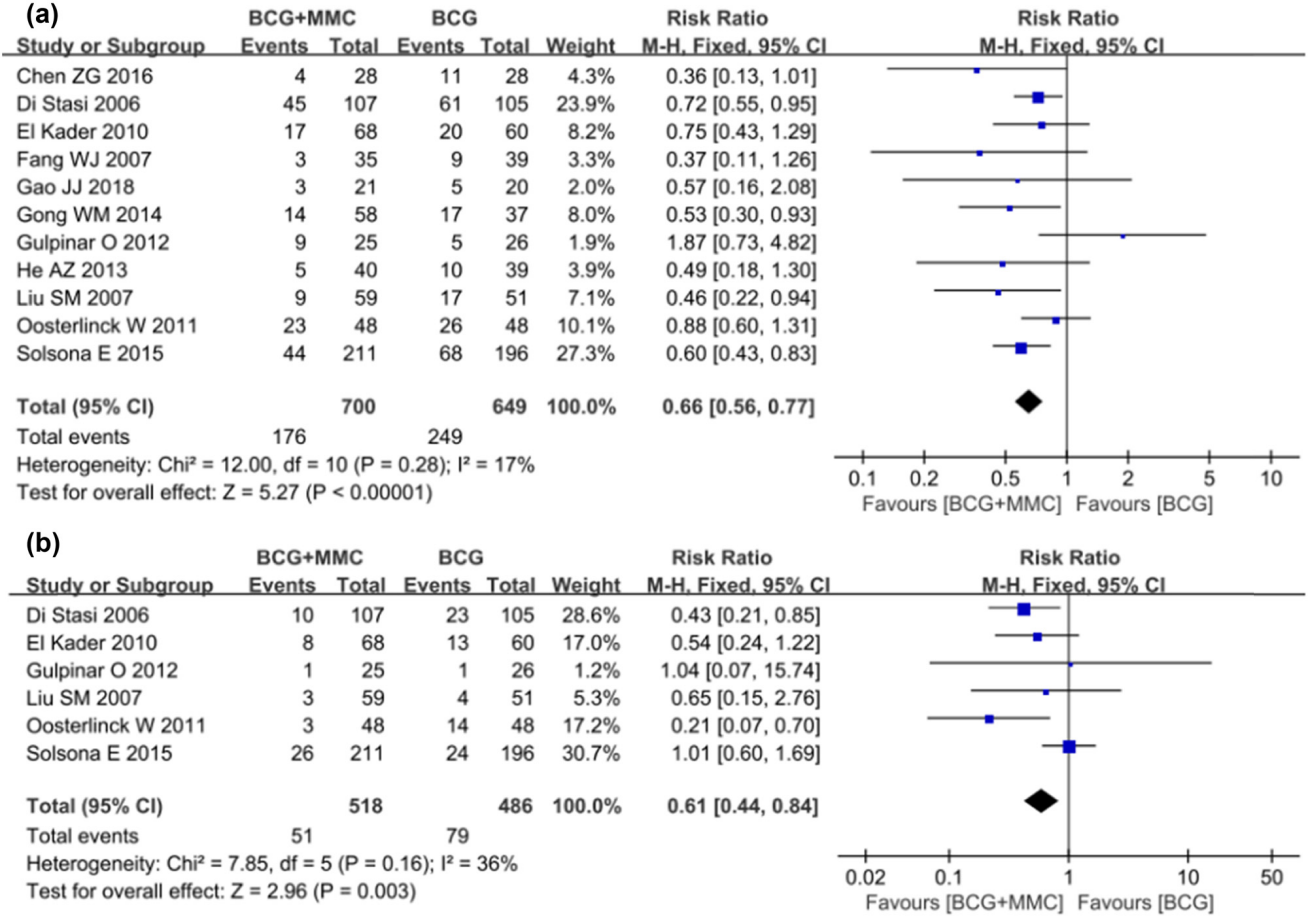
Forest plot of primary outcomes: (a) recurrence rate and (b) progression rate.

Recurrence of the disease was reported in all 11 studies. There was no heterogeneity among the studies (*I* ^2^ = 17%, *P* = 0.28). Therefore, the FEM was used for meta-analysis. The results showed that intravesical instillation of BCG combined with MMC could significantly reduce the recurrence rate of disease compared with that of BCG alone (RR 0.66, 95% CI: 0.56–0.77, *P* < 0.00001, Figure 3a).

Six studies compared the progress of the disease. There was no heterogeneity among the studies (*I* ^2^ = 36%, *P* = 0.16). The FEM was used for Meta-analysis. The results showed that compared with BCG perfusion alone, the combined group could significantly reduce the rate of disease progression (RR 0.61, 95% CI: 0.44–0.84, *P* = 0.003, Figure 3b).

### 3.4 Secondary outcomes

**Figure 4:**
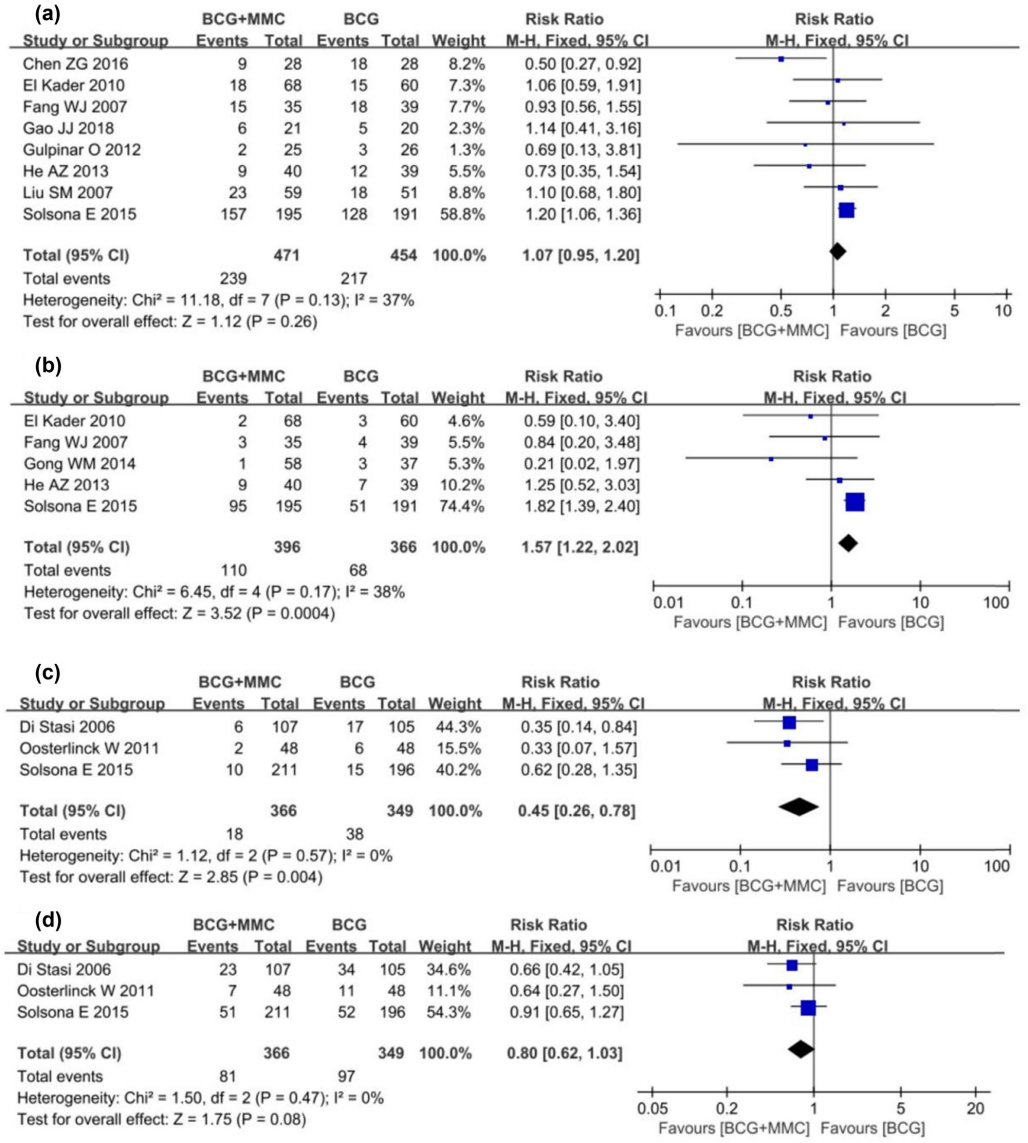
Forest plot of Secondary outcomes: (a) local adverse reactions; (b) systemic adverse reactions; (c) disease-specific mortality; and (d) all-cause mortality.

Eight studies compared the incidence of local adverse reactions in patients with intravesical instillation. The heterogeneity among these studies was low (*I* ^2^ = 37%, *P* = 0.13), so the FEM was used for meta-analysis. The results showed that there was no significant difference between BCG combined with MMC and BCG alone in terms of local adverse reactions of bladder instillation (RR 1.07, 95% CI: 0.95–1.20, *P* = 0.26, Figure 4a).

Five studies compared the incidence of systemic adverse reactions, and heterogeneity tests showed that the heterogeneity among the studies was low (*I* ^2^ = 38%, *P* = 0.17), so the FEM was used for meta-analysis. The results showed that in terms of the incidence of systemic adverse reactions, BCG combined with MMC had a higher incidence of systemic adverse reactions than intravesical instillation of BCG alone (RR 1.57, 95% CI: 1.22–2.02, *P* = 0.0004, Figure 4b).

Only three studies reported disease-specific mortality and all-cause mortality. For disease-specific mortality, the heterogeneity among the studies was low (*I* ^2^ = 0%, *P* = 0.57). The results showed that BCG combined with MMC had lower disease-specific mortality (RR 0.46, 95% CI: 0.26–0.78, *P* = 0.004, Figure 4c) than intravesical instillation of BCG alone. In terms of all-cause mortality, the heterogeneity test of the combined analysis showed that the heterogeneity among the studies was low (*I* ^2^ = 0%, *P* = 0.47). The results showed that there was no significant difference between the combined treatment group and BCG alone (RR 0.80, 95% CI: 0.62–1.03, *P* = 0.08, Figure 4d).

### 3.5 Subgroup analysis

**Figure 5:**
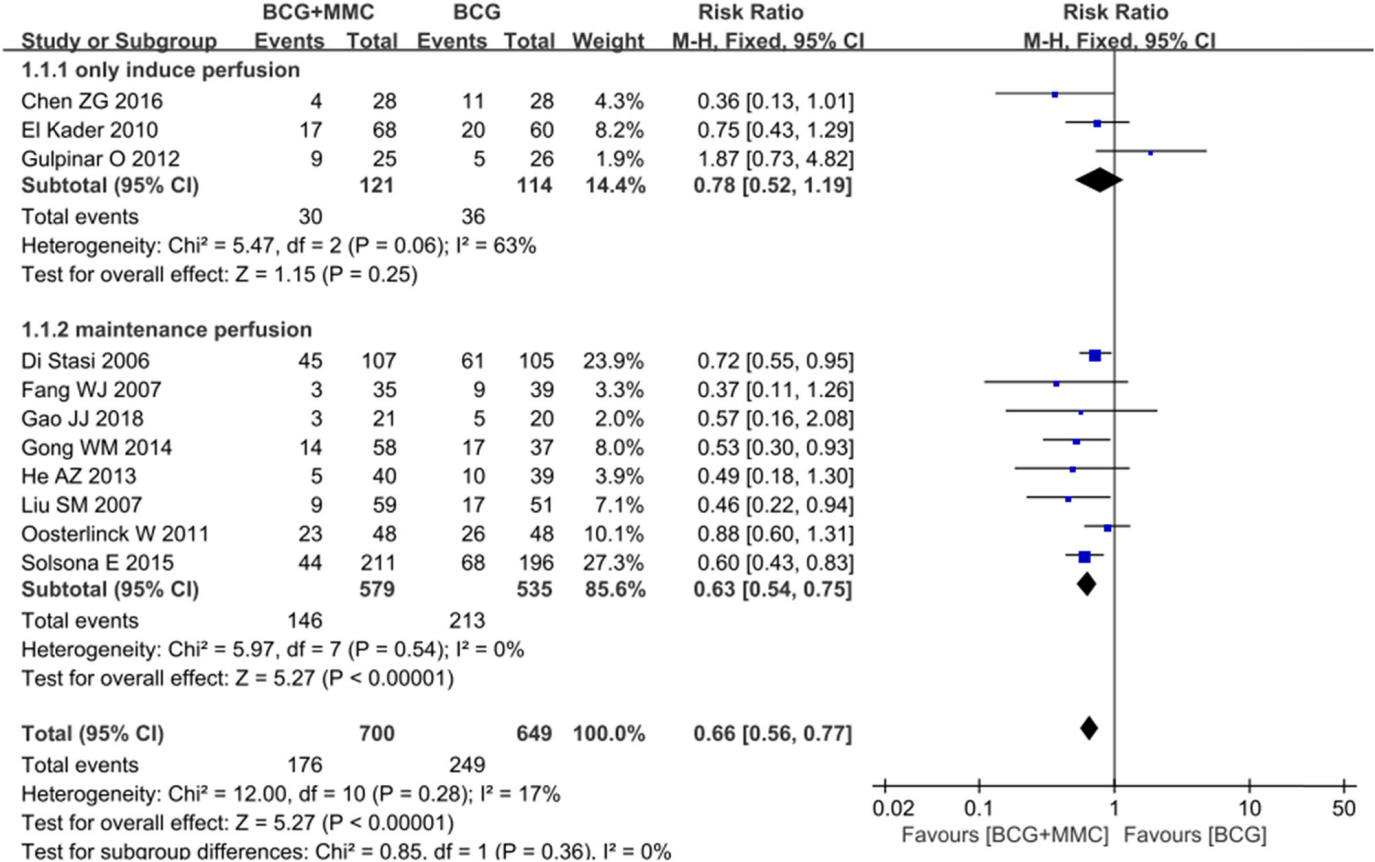
Forest plot and meta-analysis of recurrence rate between BCG combined with MMC and BCG alone: subgroup analysis based on induced perfusion and maintenance perfusion.

Each study paid more attention to the outcome indicators of disease recurrence, progression, and adverse reactions. Combined with the original study, we only made a subgroup analysis of the main outcome index of disease recurrence and were divided into subgroups according to when induced perfusion was performed and maintenance perfusion was performed. Due to the limited number of original studies on other outcome indicators such as disease progression, adverse reactions, and disease-specific death, there are no further subgroups. Three studies only performed induced perfusion, while the other eight studies performed maintenance perfusion on the basis of induced perfusion. The results of subgroup analysis showed that when only induced perfusion was performed, there was no significant difference in disease recurrence between the combined group and the BCG alone group (RR 0.78, 95% CI: 0.52–1.19 *P* = 0.25). When maintenance perfusion was performed, BCG combined with the MMC perfusion group could significantly reduce the disease recurrence rate (RR 0.63, 95% CI: 0.54–0.75, *P* < 0.00001, Figure 5).

### 3.6 Publication bias

**Figure 6:**
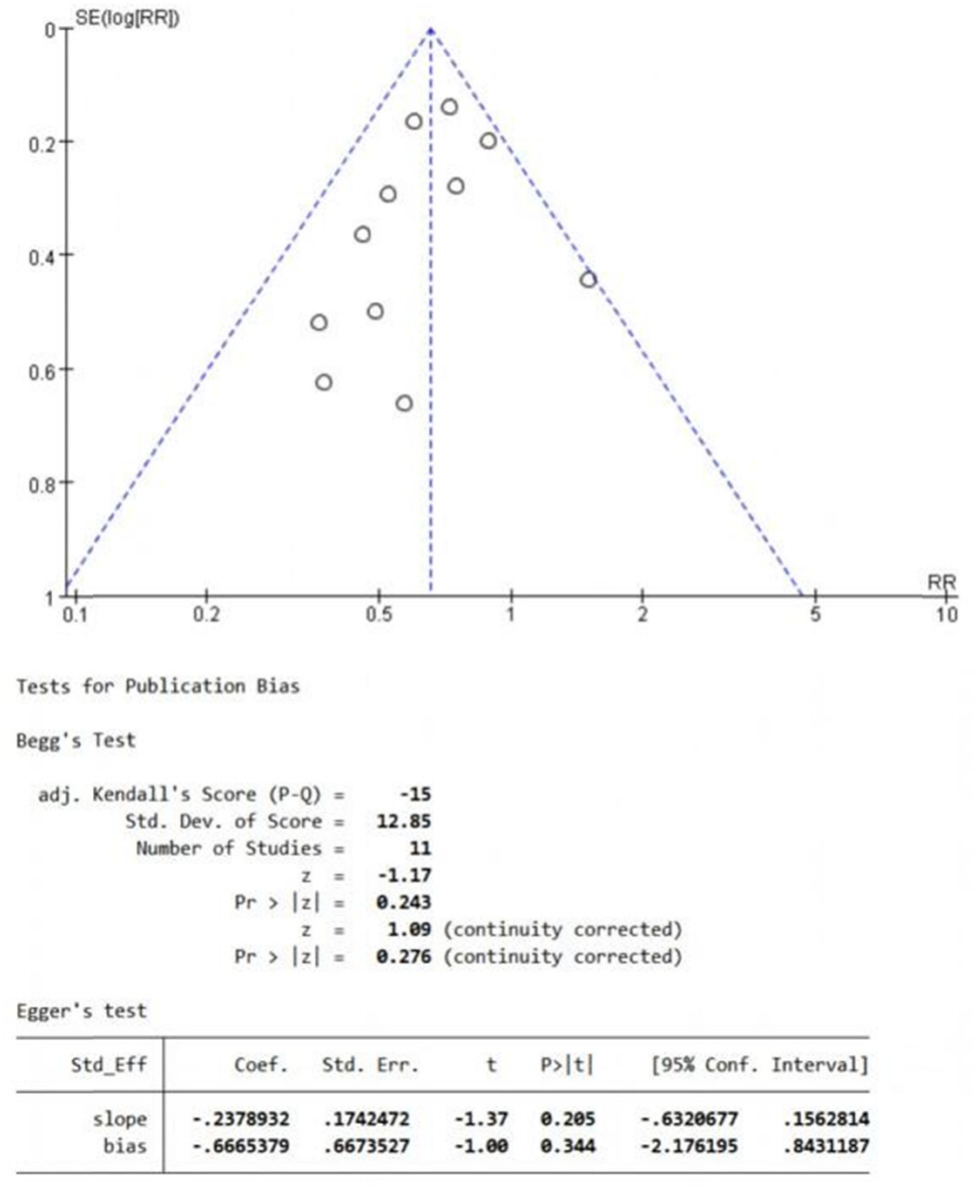
Publication bias: Funnel plot and Begg’s test and Egger’s test of the eleven studies involving the outcomes of recurrence rate.

In this study, the funnel chart of the main outcome indicator of disease recurrence was drawn by Revman5.4 software as follows. At the same time, the publication bias was quantitatively tested by Egger regression analysis and the Begg rank correlation method in Stata16.0 software. The results of the Egger regression analysis showed that the quantitative results of the Begg rank correlation method showed that the publication bias was 0.276 > 0.05. The results of the Egger regression analysis showed that the publication bias was 0.344 > 0.05 (Figure 6). The tests of both methods suggested that there was no significant publication bias in the included study.

## 4 Discussion

This meta-analysis aimed to evaluate the results of BCG combined with MMC compared with BCG alone in tumor recurrence, disease progression, adverse reactions, all-cause mortality, and disease-specific mortality in patients with NMIBC. Compared with BCG intravesical instillation alone, BCG combined with MMC had more advantages in disease recurrence, progression, and disease-specific mortality but had a higher incidence of systemic adverse reactions, and there was no significant difference in all-cause mortality and local adverse reactions between the two groups. When we conducted a subgroup analysis of whether or not to maintain perfusion, we found that there was no significant difference in disease recurrence between the two groups if only induced perfusion was used.

Since Morales first proposed BCG for bladder instillation and 6 weeks induction instillation in 1976 [6], BCG was not widely accepted not until 1980; an RCT study of the Southwest Cancer Group (SWOG) in the United States showed that BCG for bladder cancer patients after standard surgery had clear benefits in reducing the recurrence rate and increasing the median recurrence-free survival time [23]. The current guidelines recommend TURBT combined with intravesical drug instillation as the gold standard treatment for NMIBC patients, which mainly includes immunomodulator and chemotherapy drugs, but the choice of postoperative infusion drugs should be individualized according to the risk stratification of NMIBC patients [4].

Although BCG is currently the standard infusion drug for medium- and high-risk NMIBC patients, some patients still relapse or progress to MIBC. Bourlotos’s study showed that bladder instillation of BCG may cause dysuria, we hypothesized that BPH surgery before BCG instillation might improve tolerance in patients with concomitant BPH [24]. de Jong et al. identified three distinct BCG response subtypes (BRS1, BRS2, and BRS3), and the findings confirmed the prognostic relevance of molecular subtypes in high-risk NMIBC (HR-NMIBC) patients. The results showed that patients with BRS3 tumors had reduced relapse-free and progression-free survival compared with patients with BRS1/2 tumors and that BRS3 tumors were more frequently associated with Treg macrophages and B cells associated with immunosuppression, findings associated with poor clinical outcomes after BCG therapy. Thus, the identification of BRS3 tumors may be a critical step in the implementation of a more aggressive treatment regimen, such as early RC [25]. Witjes’s study demonstrated that Bladder EpiCheck holds clinical significance, exhibits a high negative predictive value, reduces the risk of false positive results on subsequent endoscopy, and avoids temporary interruption of therapy. Its use in clinical routine could reduce the number of follow-up cystoscopies, and thus associated patient and financial burdens [26]. Poli et al. analyzed the expression levels of CK20, NLRP3, NLRP4, NLRP9, and NAIP in urine sediments from patients before TURBT, 3 weeks after surgery, at the beginning of the BCG induction cycle and then before each instillation event. They found that higher levels of NLRP4 and NLRP9 were associated with an increased risk of recurrence. If larger cohort studies are conducted to confirm these results, assessing the expression levels of NLRP 4 and NLRP 9 would be useful in predicting BCG failure, playing a key role in the decision-making process of early radical surgical intervention [27].

A study of BCG maintenance perfusion therapy for Ta and T1 by Cambier showed that the 1-year recurrence rate was 25.9% (95% CI: 23.8–27.9), the 5-year recurrence rate was 41.3% (95% CI: 39.0–43.7%), and the progression rate was 9.5% 13.4% [8]. BCG-mediated tumor immunity includes three steps: (1) BCG attachment, (2) BCG cell internalization, and (3) BCG-mediated initiation of related innate and adaptive immune responses [28]. It can be seen that BCG attachment is the first and crucial step for it to exert its anti-tumor effect. The results of an *in vitro* study of Kavoussi showed that the chemical destruction of urinary tract epithelium induced by MMC can make BCG adhere to the bladder wall more effectively, thus improving the immune response and anti-tumor activity. Moreover, the instillation of MMC can also promote the uptake of BCG by the bladder wall and activate related immune effector cells [29]. In addition, Matsushima through an *in vitro* study of Tis found that MMC combined with BCG treatment can inhibit tumor growth and cell proliferation and prolong survival compared with BCG alone [30]. Intravesical instillation of BCG combined with MMC can increase the anti-tumor effect in patients with NMIBC, and it is logical whether chemotherapy and immunotherapy with two different anti-tumor mechanisms are more effective than BCG alone.

A meta-analysis study by Houghton showed that the combination of chemotherapy on the basis of maintenance therapy did not significantly reduce the recurrence rate (RR 0.92, 95% CI: 0.79–1.99, *P* = 0.32) or the progression rate (RR 0.88; 95% CI 0.61–1.27, *P* = 0.5) [31]. However, there are few original studies included in this study, and there are only two studies combined with BCG and MMC. However, a meta-analysis study by Lan showed that the recurrence rate of NMIBC patients treated with BCG combined with MMC was significantly lower than that of BCG or MMC alone (RR 0.81, 95% CI: 0.72–0.92, *P* < 0.001), but there was no significant difference in disease progression rate, all-cause mortality, and disease-specific mortality. At the same time, they conducted a subgroup analysis of MMC perfusion alone and BCG perfusion alone in terms of disease recurrence, and the results showed that the combined group had a lower rate of disease recurrence than BCG alone [32], which was consistent with our results. Because their control group contained both BCG and MMC, we did not further compare the results in other aspects such as disease progression and adverse reactions.

Our meta-analysis had some potential limitations. Although we have strict inclusion and exclusion criteria, like the meta-analysis of most drug trials, eleven RCTs included in this meta-analysis are different in treatment regimens, BCG and MMC doses, BCG strains, disease risk stratification, specific treatment time and follow-up time, and geographical distribution, which may be the causes of heterogeneity. For BCG strains, a study by Witjes compared the efficacy of BCG Connaught and BCG TICE and showed that BCG Connaught had a lower recurrence rate than BCG TICE when only induced perfusion, but when perfusion was maintained, the result was the opposite [33]. Because the original study is based on published results, we cannot obtain data from individual patients, and only a small number of studies have mentioned BCG strains and risk stratification of NMIBC patients in the included studies, we have not been able to conduct further subgroup analysis.

## 5 Conclusions

This study shows that compared with BCG alone, intravesical instillation of BCG combined with MMC can reduce the disease recurrence rate, disease progression rate, and disease-specific mortality in patients with NMIBC. However, there is a higher incidence of systemic adverse reactions. There was no significant difference in the incidence of all-cause death and local adverse reactions between the two groups. Due to the limitation of the number of studies included and the different doses, strains, and risk stratification of NMIBC patients in different studies. Multicenter, high-quality, more rigorous trial design studies are needed to further explore the effectiveness and safety of combined perfusion in the future.
